# Remote workers’ life quality and stress during COVID-19: a systematic review

**DOI:** 10.1093/eurpub/ckae167

**Published:** 2025-02-06

**Authors:** Elisabetta Carraro, Paola Rapisarda, Daniela Acquadro Maran, Sofia Filippetti, Marco Palella, Eliana Pellegrino, Margherita Ferrante, Giuseppe La Torre, Maria Fiore

**Affiliations:** Department of Public Health and Pediatric Sciences, University of Turin, Italy; Department of Medical, Surgical and Advanced Technologies “G.F. Ingrassia”, University of Catania, Italy; Earth and Environmental Sciences Ph.D. Course of Department of Biological, Geological and Environmental Sciences, University of Catania, Italy; Department of Psychology, University of Torino, Italy; Department of Public Health and Pediatric Sciences, University of Turin, Italy; Department of Medical, Surgical and Advanced Technologies “G.F. Ingrassia”, University of Catania, Italy; Department of Medical, Medical Specialization School in Hygiene and Preventive Medicine, Surgical Sciences and Advanced Technologies “G.F. Ingrassia”, University of Catania, Italy; Department of Medical, Surgical and Advanced Technologies “G.F. Ingrassia”, University of Catania, Italy; Department of Medical, Surgical and Advanced Technologies “G.F. Ingrassia”, University of Catania, Italy; Department of Public Health and Infectious Diseases, Sapienza University of Rome, Italy; Department of Medical, Surgical and Advanced Technologies “G.F. Ingrassia”, University of Catania, Italy

## Abstract

COVID-19 pandemic led to the adoption of a different working approach: “The remote working.” Evidence about the association of remote working with stress outcomes and life quality is lacking. This systematic review provides an overview of the effects of COVID-19 pandemic on remote-workers’ stress and life quality. We conducted systematic literature searches in databases including Pubmed, Scopus and Web of science, from September 2020 to September 2023. Screening of titles, abstracts, and full texts were performed according to the Preferred Reporting Item for Systematic Review and Meta-analyses. The quality of the included studies was assessed using the Newcastle-Ottawa Scale. The review highlighted possible predictors (work-family conflict or a condition of social isolation) associated with improvement or worsening of quality of life and stress. The results highlighted the association between stress and family difficulties (β: −0.02, *P*-value <0.05), isolation during the first (β: −0.22, *P*-value <0.05) and second pandemic waves (β: −0.40, *P*-value <0.05) or due to the advancing age of workers (β:0.19, *P*-value <0.05) and (β: −0.05, *P*-value <0.05), furthermore some job categories presented greater stress such as teachers (16.94 ± 5.46). Conversely, remote working positively affected life quality, enhancing factors such as creativity (Average Variance Extracted, AVE: 0.41, *R*^2^: 0.17) and self-efficacy (AVE: 0.60, *R*^2^: 0.36). Future research should focus more on the relationship between work and family and on interventions that counteract social isolation.

## Introduction

On 11 March 2020, the World Health Organization (WHO) declared COVID-19 a pandemic [1] and urged countries to “take urgent and aggressive action” [2]. The pandemic posed significant challenges for workplaces, requiring protective measures and a rethinking of traditional work patterns to balance work, health, and safety. Remote working became a key solution to meet these demands.

Şentürk *et al*. [3] suggest that remote working may impact workers’ stress levels and quality of life. To our knowledge, no systematic reviews exist on this topic. This review examines observational studies exploring the relationship between remote working, stress levels, and quality of life, highlighting limitations and offering recommendations for future research.

## Methods

### Data sources and search strategy

This systematic review was strictly reported based on the Preferred Reporting Items for Systematic Review and Meta-analyses (PRISMA) statement [4, 5]. The protocol of the present study was registered in the international prospective register of systematic review “PROSPERO” (registration number CRD42022370868). The protocol was not published in any peer-reviewed journal. We searched papers using PubMed, Scopus, and Web of Science. We have filtered only research articles published in English language and selected the keywords reported in the Supplementary Appendix SA. We located all the relevant keywords for the topic by background reading, identifying different spellings, tenses, and word variants of keywords, synonyms, and related concepts. Reference lists of selected studies were checked to ensure complete coverage.

### Inclusion/exclusion criteria

We included studies carried out in the period September 2020–September 2023, only in English language, studies with an observational design (longitudinal and cross-sectional studies), articles including the use of “remote working” during the pandemic period and including stress and/or quality of life outcomes. Studies including reviews, conference proceedings, editorials, articles without statistical data and those published beyond the established period were excluded.

### Data extraction

Two authors (P.R. and M.P.) independently reviewed all retrieved articles and extracted data. Initially, titles and abstracts were screened to identify potentially eligible studies, followed by full-text review to confirm their inclusion in this systematic review. For each included study, the following data were extracted: first author, publication year, country, study design, data collection period, target population, age (in years), sample size, study aim, outcome measures (questionnaire), and results. The extracted data were cross-checked, and any disagreements were resolved through discussion or consultation with a third author (M.F.).

### Quality assessment

The methodological quality of the included studies was evaluated by two independent researchers (P.R. and M.P.) using the Newcastle-Ottawa Scale (NOS) star system (range from 0 to 10 stars), which focuses on three broad perspectives: the selection of the study groups, the comparability of the groups, and the ascertainment of either the exposure or outcome of interest [6]. The number of stars is positively associated with the quality of the study. According to the scoring algorithms, a score ≥7 was considered as “good.” Any disagreement between the two authors was resolved by a consensus session with a third author (M.F.).

## Results

**Figure 1.**
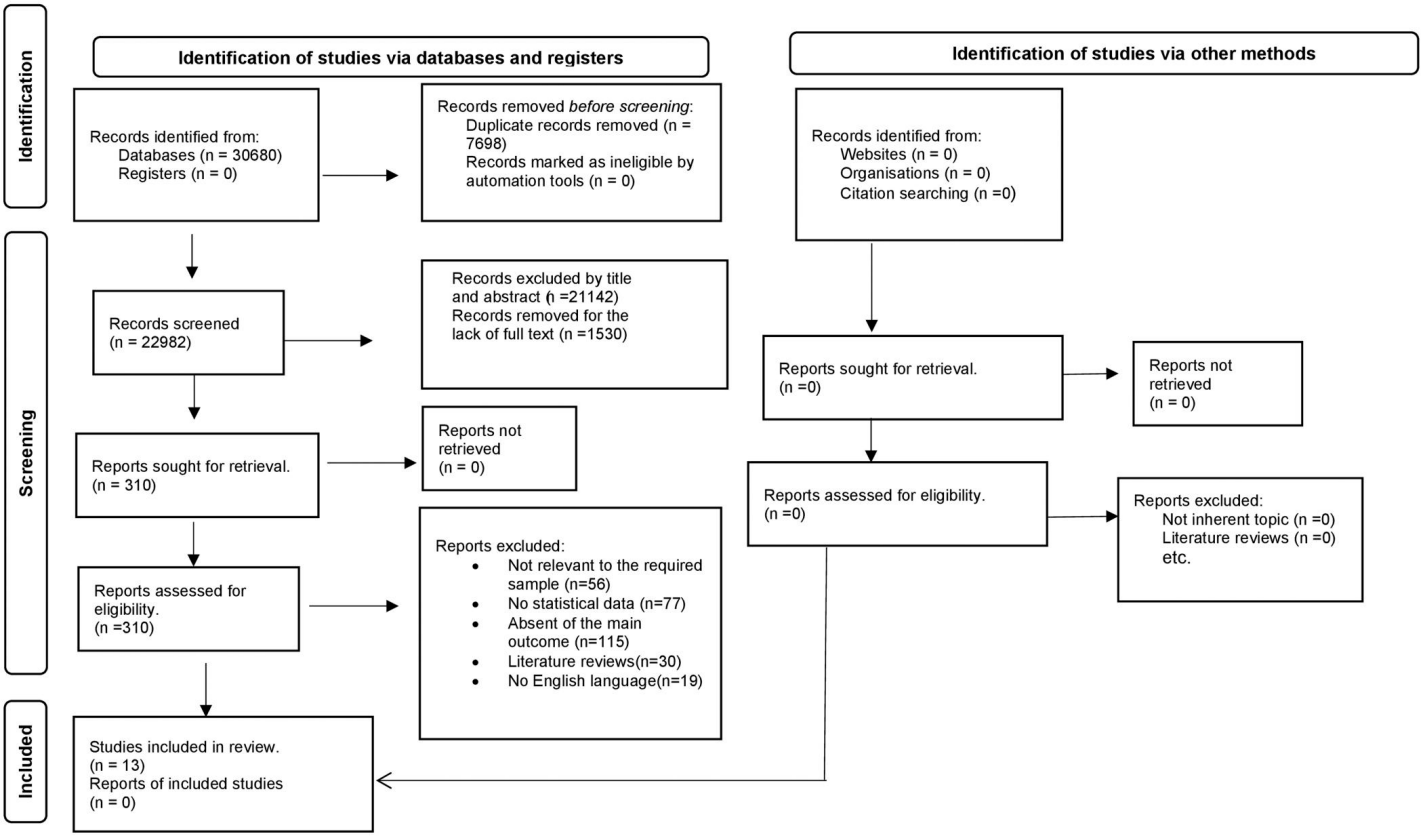
Flow chart of studies’ identification and selection [4, 5].

The general characteristics of the included studies are reported in Table 1. We found a total number of 30 680 articles. From which, 7698 were excluded because of duplicate records. The remaining 22 982 articles have been evaluated by title and abstract and 21 142 articles were excluded, while 1530 articles removed for the lack of full text. The remaining 310 articles were controlled and checked the full text and 297 were excluded for the following causes: 56 because they did not take into account subjects who had used remote work during the pandemic period, 77 for the absence of the statistical data, 115 for the absence of the main outcome, 30 because were literature reviews, and 19 because not published in English language. Finally, 13 articles have been included in this systematic review [3, 7–18]. The full process of article collection, screening, and eligibility assessment is presented in Fig. 1. In particular, of the 13 selected studies, nine refer to stress [3, 7–14], two to the quality of life [15, 16], and the last two deal with both the main outcomes [17, 18]. The country analysis shows that most studies on these topics were conducted in Europe, namely Italy (4/13), Poland (2/13), Turkey (1/13), and the United Kingdom (1/13). One study was performed in North America (USA) and another in South America (mainly Colombia and Ecuador). One study was conducted in Hong Kong, one in Korea, and another in Australia.

### Quality assessment

Overall, the included studies rated from 7 to 10 stars (Supplementary Appendix SB). In particular, 3 out of 13 articles had a score of 7 [14, 16, 17]; 4 out of 13 articles had a score of 8 [3, 10, 13, 15]; and 6 out of 13 had a score of 9 [7–12, 18].

**Table 1.** General characteristics of the included studies

Author, publication year	Country	Study design and data collection period	Target population	Age (years)	Sample size	Study aims
B. Barbieri, 2021	Cagliari (Italy)	Web based survey July 2020	Smart workers during the Covid19 pandemic “great lockdown”	45.1 ± 7.8 (Mean± SD)	293 (216 *♀*/77 *♂*)	Impact of job demands, organizational job and personal resources on workers' quality of life considering the potential mediating role of job satisfaction and perceived stress
B. Barone Gibbs, 2021	Pittsburgh (USA)	Prospective survey Baseline: *January 2018* Follow up: *May–June 2020*	Different categories of desk workers	45.4 ± 12.3 (Mean± SD)	112 (77 *♀*/35 *♂*) Loss to follow up 22 participants	Longitudinal impact of COVID-19 restriction on desk workers' work practices, lifestyle and well-being
T. Galanti, 2021	Bologna (Italy)	Cross-sectional *May–July 2020*	Public and private organizations full time smart-workers	49.8 ± 9.4 (Mean± SD)	209 (149 *♀*/60 *♂*)	Family-work conflict, social isolation, distracting environment, job autonomy, and self-leadership on employees’ productivity, work engagement and stress experienced during the pandemic
M. Graham, 2021	Bundoora (Australia)	Cross-sectional *March–April 2020*	Part-time and Full-time smart workers	18–35; 36–45; 46–55; 56 and over (Age class)	658 (499 *♀*/159 *♂*)	General health, pain, stress, work-family, family-work conflict, gender differences and parental responsibilities of working at home
T. D. Jakubowski, 2021	Katowice (Poland)	first stage: Retrospective study *September–October 2020 *second stage: cross-sectional study *December 2020–February 2021*	Teachers of moving education in primary and secondary schools, by the virtual space	43.76 ± 8.31 (Mean± SD)	285 - **first stage:** 145 (130 *♀*/15 *♂*) - **second stage:** 140 (121 *♀*/19 *♂*)	Association between distance education and teachers’ well-being during the COVID-19 pandemic.
A. Lipert 2021	Lodz (Poland)	Web based survey *1–14 April 2020*	General population by working modes: -workplace working -remotely working -Nonworking	18–65 (Age class)	1959 (1681 *♀*/278 *♂*)	Stress and sleep quality relationship by physical activity during COVID-19 pandemic lockdown by different working modes
E. Mari, 2021	Rome (Italy)	Web-based survey *April 2020*	Practitioners (lawyer, psychologist, accountant etc.), managers, executive employees, teachers	42.3 ± 10.5 (Mean± SD)	628 (489 *♀*/139 *♂*)	Psychological variables differences by different professionist groups
J. Sandoval-Reyes, 2021	South America (mostly Colombia and Ecuador)	Web-based survey *April–May 2020*	General population	Mean 29.1 (SD not reported)	1285 (847 *♀*/438 *♂*)	Relationship between remote work, work stress and work–life developed during pandemic; Impact on work productivity, satisfaction and work-life balance during the COVID-19 pandemic
E. Şentürk, 2021	Istanbul (Turkey)	Cross-sectional and Web-based survey *25 October–24 December 2020*	Different categories of remote workers	35.6 ± 6.8 (Mean ± SD)	459 (205 *♀*/254 *♂*)	Predictors of depression, anxiety and stress Work and home life changes during the COVID-19 pandemic by sex
R. Truzoli, 2021	Milan (Italy)	Web-based survey *April–May 2020*	High school Teachers	49.8 ± 10.1 (Mean ± SD)	107 (69 *♀*/38 *♂*)	Relationship between some protective (e.g. locus of control) and risk factors (e.g. stress) on satisfaction levels
V.G. Girish, 2022	Daejeon (Republic of Korea)	Web based survey *Not reported*	Employees	20–29; 30–39; 40–49; 50+ (Age class)	385 (226 *♀*/159 *♂*)	Association between smart working and employees’ quality of life
K. Platts, 2022	United Kingdom	Cross-sectional *May–August 2020*	Employees	16–24; 25–34; 35–44; 45–54; 55+ (Age class)	623 (234 *♀*/384 *♂*/5 missing)	Relationship between the enforced home working and employee’s wellbeing by different stress markers
A.M.Y. Chu, 2022	Hong Kong (Hong Kong)	Web-based survey *September 2020*	Full-time employees	18–24; 25–34; 35–44; 45–54; 55–64; 65 and over (Age class)	500 (288 *♀/*212 *♂*)	Effect of company support, supervisor’s trust in the subordinate and work-life balance on stress and happiness

### Job stress

The studies included in the review, despite having investigated the same outcome, used different questionnaires, which made it difficult to group them together. Therefore, we present below a synthesis of each study results (Table 2).

**Table 2.** Confounders, outcomes and results of the included studies

Author, publication year	Confounders	Outcome measured (Questionnaire)	Results
**B. Barbieri, 2021**	Female, Married, University degree	**Quality of life** **Exogenous variables** Job demand (social isolation, workload); Organizational job resources (perceived organizational support); Personal resources (self-efficacy, vision about future, commitment to organizational change) **Mediating variables** job satisfaction (Brief overall job satisfaction measure II) and perceived stress (Perceived stress scale IPSS-10)	**Mean ±SD; Min; Max** Quality of life 0.00 ± 1.99; −6.95; 4.55 Overall model exogenous variables impact *R*^2a^: 73.9 (76.0% for *♀*) Quality of life *R*^2^: 56.9 (58.5% for *♀*) Mediating variables impact *R*^2^: Job satisfaction 37.0% (41.5% for *♀*) and Perceived stress 55.3% (58.9% for *♀*)
**B. Barone Gibbs, 2021**	Ethnicity, Gender, Physical activity, Race	**Quality of life** (Short Form (SF-36)) **Stress** [Health and Work Questionnaire (HWQ)]	**Before Covid-19 shelter-at-home *vs* During shelter-at-home;** mean ± SD; *P*-value ***Quality of life*** General health (70.5 ± 15.1 *vs* 69.9 ± 16.5) NS; Physical functioning(92.1 ± 14.5 *vs* 91.7 ± 16.0) NS; Role limitations due to physical health (93.7 ± 22.5 *vs* 88.1 ± 26.4) NS; Pain (87.1 ± 14.7 *vs* 81.7 ± 18.1) <0.00; Emotional well-being (77.5 ± 14.8 *vs* 71.4 ± 17.9) <0.00; Social functioning (90.5 ± 17.4 *vs* 84.1 ± 19.4) <0.01; Role limitations due to emotional health (87.4 ± 26.2 *vs* 74.8 ± 36.2) <0.00; Energy/fatigue (57.6 ± 17.9 *vs* 54.5 ± 19.6) <0.05. ***Health and Work Questionnaire*** Stress (single item) (4.9 ± 2.5 *vs* 4.7 ± 2.7) NS.
**T. Galanti, 2021**	Children less than 14 years old,	**Stress** (Weinert *et al*. Questionnaire^k^)	**Mean ±SD** Stress 2.45 ± 1.19 **Stress β (SE)** family-work conflict 0.31 (0.06) (*P* < 0.01) social isolation 0.48 (0.06) (*P* < 0.01) distractive W. Env. 0.05 (0.06) job autonomy 0.03 (0.07) self-leadership −0.03 (0.09)
**M. Graham, 2021**	Gender, Presence of children	**Stress** [Copenhagen Psychosocial Questionnaire (COPSOQ)]	**GLM (β), 95% CI *Stress*** *♂ (ref) vs ♀* Effect of sex (unadj.): 0.26 (0.13, 0.39) (*P* < 0.01) Effect of sex (adj): 0.13 (–0.00, 0.27) *Having no children (ref) vs having children* Effect of having children (unadj.): 0.05 (–0.07, 0.17) Effect of having children (adj): 0.04 (–0.09, 0.18) *♂with children (ref) vs ♂without children—♀with children—♀without children* Effect of sex (*♂* ref.) and Children Present During Time Spent Working From Home (unadj.) *♂*with children *vs ♂*without children 0.08 (−0.15, 0.31) *♂*with children *vs ♀*with children .39 (0.18, 0.61) (*P* < 0.01) *♂*with children *vs ♀*without children 0.26 (0.07, 0.46) (*P* < 0.01) Effect of sex and Children Present During Time Spent Working From Home (adj) *♂* with children *vs ♂*without children 0 .03 (−0.21, 0.26) *♂*with children *vs ♀*with children 0.24 (0.04, 0.45) (*P* < 0.05) *♂*with children *vs ♀*without children 0.10 (−0.09, 0.30)
**T. D. Jakubowski, 2021**	Total number of children	**Stress** [The Depression Anxiety & Stress Scales-21 (DASS-21)]	**Stress correlation by independent variables** **1st COVID-19 pandemic wave *vs* 2nd COVID-19 pandemic wave** ***Sperman's rank or Pearson's correlation coefficient*** A) Age: 0.20 (*P* < 0.05) *vs* −0.06 B) Years of work as a teacher: 0.13 *vs* −0.05 C) Total n. of children: 0.27 (*P* < 0.05) *vs* −0.06 D) n. of children up to 8 years old: −0.15 *vs* −0.06 E) n. of children 9–15 years old: 0.08 *vs* −0.01 F) n. of children 16–19 years old: 0.24 (*P* < 0.01) *vs* 0.04 G) Relationship quality : −0.29 (*P* < 0.01) *vs* −0.29 (*P* < 0.01) H) Social relation quality : −0.22 (*P* < 0.01) *vs* −0.40 (*P* < 0.001) I) General social support: −0.11 *vs* −0.24 (*P* < 0.01) L) Emotional social support: −0.09 *vs* −0.25 (*P* < 0.01) M) Instrumental social support: −0.09 *vs* −0.20 (*P* < 0.05) N) Relationship satisfaction: −0.09 vs −0.25 (*P* < 0.01) O) Perceived injustice: data not furnished *vs* −0.36 (*P* < 0.001) P) Blame/unfairness: data not furnished *vs* 0.58 (*P* < 0.001) Q) Severity/irreparability: data not furnished *vs* 0.63 (*P* < 0.001) **The variable E (1st wave): Crude. R^2^= 6%** **The variables G, H, P, Q and gender (2nd wave): Crude. *R*^2^ = 47%** **Mean stress by sex** mean ± SD; *P*-value, effect size^b^ *♀vs ♂* 1st COVID-19 pandemic wave 14.9 ± 10 *vs* 16.8 ± 12 *P*-value 0.72, effect size 0.19 2nd COVID-19 pandemic wave 16.2 ± 11 *vs* 9.8 ± 8 *P*-value 0.02, effect size 0.59
**A. Lipert 2021**	Physical activity during pandemic	**Stress** [Perceived Stress Scale (PSS)]	**Overall level of stress (Mean ± SD)** Working in the workplace 21.5 ± 7.1 Working remotely 21.5 ± 7.2 Nonworking 22.6 ± 7.5 (*P* < 0.01) **Frequency of study population by work modes and stress categories (%)** *Working in the workplace* low = 13% moderate = 53% High = 34% *Working remotely* low = 15% moderate = 61% High = 24% *Nonworking* low = 14% moderate = 63% High = 24%
**E. Mari, 2021**	Gender, Geographic Area	**Stress** [Perceived Stress Scale (PSS)]	**Mean ±SD;** (ANOVA, *P*-value 0.06) Teachers 16.94 ± 5.46 Practitioners 15.89 ± 5.35 Managers 15.17 ± 5.39 Executive employees 16.27 ± 6.23
**J. Sandoval-Reyes , 2021**	Not Reported	**Stress** [Work Stress Questionnaire (Folkman and Lazarus’s, 1985)]	**Structural model Path coefficient; 95% CI; t-value** 1. (Remote work demands → Work stress → Work life balance) −0.05; (−0.07, −0.04); 6.29 2. (Remote work demands → Work stress → Work Productivity) −0.05; (−0.07, −0.04); 5.72 3. (Remote work demands → Work stress→ Job satisfaction) −0.09; (−0.11, −0.07); 8.19 4. (Remote work demands → Work stress → Job engagement) −0.059; (−0.080, −0.041); 5898
**E. Şentürk, 2021**	Educational status, Having a child, Gender	**Stress** [Depression Anxiety Stress Questionnaire—Short Form (DASS-21)]	**Prevalence of stress:** Normal 369 (80.4%) Mild 89 (19.4%) Moderate 1 (.2%) Severe 0 (0%) ***Coef.* multiple linear regression (95% CI) and *P*-value of “stress”** Sex 0.15 (0.64, 1.99) ; < 0.00 Age −0.04 (–0.08, 0.03); 0.43 Educational status −0.02 (–0.92, 0.46); 0.508 Working organization 0.03 (–0.52, 1.30); 0.40 Having a child 0.08 (–0.33, 1.62); 0.19 Changes in time spent on household chores 0.01 (–0.17, 0.21); 0.85 Changes in time spent on childcare −0.02 (−0.29, 0.17); 0.61 Changes in daily working hours 0.03 (–0.10, 0.21); 0.50 Changes in workland 0.07 (–0.12, 0.75); 0.16 Control over working hours 0.08 (–0.01, 0.96); 0.06 Distraction while working 0.09 (–0.04, 0.68); 0.08 Trouble focusing at work 0.16 (0.29, 0.92); < 0.00 Current financial state –0.03 (–0.24, 0.11); 0.48 Financial concern –0.10 (–0.37, –0.01); 0.04 Workplace loneliness –0.09 (–0.91, – 0.08); 0.02 Jenkins sleep score 0.32 (0.21, 0.35); < 0.00 Leisure-Time Exercise Questionnaire score –0.01 (–0.02, 0.01); 0.88
**R. Truzoli, 2021**	Gender, School education	**Stress** (Quick stress assessment^d^)	**Stress** ***Mean ± SD*** Overall sample: 14.9 ± 7.2 *♂*: 14.7 ± 7.8 *♀*: 15.0 ± 6.9 ***Spearman's coeff; P-value*** Stress *vs* Test efficacy scale −0.36; < 0.00 Stress *vs* Anxiety test 0.65; <0.00 Stress *vs* Depression test 0.78; <0.00 Stress *vs* Locus of control test 0.39; <.0.00
**V.G. Girish, 2022**	Education, Gender,	**Quality of life** (Five items adopted from Bai *et al*. 2017 and Oh *et al*. 2011^e^^,^^f^)	**Quality of life** (results of measurement model by Confirmatory Factor Analysis) *λ* ^c^ *; Mean ± SD* I have a happy life 0.851; 3.84 ± 0.71 I am living a worthy life 0.87; 3.84 ± 0.71 I am proud of my life 0.83; 3.83 ± 0.79 My future is bright 0.85; 3.81 ± 0.79 Overall, I am satisfied with my life 0.85; 3.86 ± 0.74 ***Quality of life*** **Average variance extracted^g^ (squared correlation coefficient)** Quality of life *vs* Communication/collaboration: 0.3 (0.09) Quality of life *vs* Work efficiency: 0.35 (0.12) Quality of life *vs* Autonomy: 0.33 (0.11) Quality of life *vs* Fairness in appraisal: 0.28 (0.08) Quality of life *vs* Work–life balance: 0.31 (0.10) Quality of life *vs* Self-efficacy: 0.60 (0.36) Quality of life *vs* Workplace creativity 0.42 (0.17) Quality of life *vs* Job satisfaction 0.57 (0.33) Quality of life *vs* Quality of life 0.82
**K. Platts, 2022**	Gender	**Wellbeing** (main outcome) measured through different stress markers [Copenhagen Psychosocial Risk Assessment Questionnaire-COPSOQIII)]	**Overall Mean** Stress: 39 Somatic Stress: 26 Cognitive Stress: 31
**A.M.Y. Chu, 2022**	Educational level, Gender,	**Effects of three stress relievers** (company support, supervisor's trust in subordinate and work-life balance) **on “psychological Well-being”** (stress and happiness). **Stress** (Questionnaire includes three items to measure level of stress: sleep qualty,^g^ loss of energy^h^ and depressed mood^i^). **Happiness** (Questionnaire adopted by Chaiprasit and Santidhirakul^j^).	**Correlation coefficient** Stress *vs* company support 0.01 Stress *vs* supervisor trust −0.01 Stress *vs* work life balance −0.22 Stress *vs* stress 0.71 Stress *vs* happiness −0.16 Stress *vs* non-work-related activities 0.63 Stress *vs* participant's work productivity −0.18 **Mean ± SD** ***Stress*** Sleep quality: 3.31 ± 1.76 Loss of energy: 3.26 ± 1.75 Depressed mood: 3.28 ± 1.67 ***Happiness*** Feed joy at work 4.92 ± 1.58 Satisfied with work 4.47 ± 1.52 Enthusiastic at work 4.01 ± 1.53

a R-squared (*R*^2^): is a statistical measure that represents the proportion of the variance for a dependent variable that’s explained by an independent variable in a regression model. If *R*^2^ of a model is 0.50, then approximately half of observed variation can be explained by the model’s inputs.

b (Hedges’ g: 0.2 = Small effect size, 0.5 = Medium effect size 0.8 = Large effect size)

c λ: Standardized factor loadings can range from −1 to 1. Loadings close to −1 or 1 indicate that the variable strongly influences the factor. Loadings close to 0 indicate that the variable has a weak influence on the factor

d Tarsitani L., Biondi l.: Sviluppo e validazione della scala VRS—Valutazione Rapida dello Stress. Med psycos, 44:163-177, 1999.

e Oh, J.H., Kim, C.W. and Choi, J.R. (2011), “A study on job satisfaction and QOL of tour guides: the case of Thailand,” International Journal of Tourism and Hospitality Research, Vol. 25 No. 3, pp. 285-304.

f Bai, L.Z. and Han, J.S. (2017), “The impact of tourists’ experiences on subjective happiness, psychological happiness and quality of life of healing tourism: based on experience economy theory,” Korean Journal of Hospitality & Tourism, Vol. 26 No. 3, pp. 1-17.

g Knudsen HK, Ducharme LJ, Roman PM. Job stress and poor sleep quality: Data from an American sample of full-time workers. Soc Sci Med. 2007 May [cited 2021 Nov 24]; 64(10):1997–2007.

h Kjellberg A, Toomingas A, Norman K, Hagman M, Herlin RM, Tornqvist EW. Stress, energy and psychosocial conditions in different types of call centres. Work. 2010; 36(1):9–25.

i Wang J, Patten SB. Perceived work stress and major depression in the Canadian employed population, 20–49 years old. J Occup Health Psychol. 2001; 6(4):283–9.

j Chaiprasit K, Santidhiraku O. Happiness at Work of Employees in Small and Medium-sized Enterprises, Thailand. Procedia—Soc Behav Sci. 2011 January 1; 25:189–200.

k Weinert C, Maier C, Laumer S. Why are teleworkers stressed? An empirical analysis of the causes of telework-enabled stress. Proc der 12 Int Tagung Wirtschaftsinformatik; 2015:1407–1421.

Barone *et al*. [18] conducted a prospective study with a sample of 112 participants (77 women and 35 men) to examine the longitudinal impact of COVID-19 on workers and evaluated stress by the Health and Work Questionnaire (HWQ) before COVID-19 pandemic (4.9 ± 2.5) and during the pandemic phase (4.7 ± 2.7). No difference was found in the perceived stress score between the two phases.

The study by Galanti *et al*. [7] used a cross-sectional design with 209 participants, including 149 women and 60 men. The aim was to evaluate the relationship between work-from-home (WFH) engagement, productivity, and stress levels. According to the JD-R model. The stress during work-from-home (WFH) was measured using the four items previously adopted by Weinert *et al*., aimed at evaluating workers’ perception of exhaustion and fatigue due to telework. Items included, e.g. the statement: “I feel exhausted after working from home” [19] Galanti *et al*. [7] explored family-work conflict, social isolation, distracting environment, job autonomy, and self-leadership (independent variables) on employees’ productivity, work engagement, and stress experienced during the pandemic. They found that stress had a moderate (β = 0.31, *P* < .01) and strong (β = 0.48, *P* < .01) positive correlation with family-work conflict and social isolation, respectively. In contrast, no correlation was found for “distracting environment” (β = 0.05, *P* > 0.05), “job autonomy” (β = 0.03, *P* >.05), and “self-leadership” (β = −0.03, *P* > 0.05).

The cross-sectional study of Graham *et al*. [9] examined the impact of working at home on stress using the Copenhagen Psychosocial Questionnaire (COPSOQ) focusing on gender and parental responsibilities differences. The study had a sample size of 658 participants, including 499 women and 159 men. Comparing women and men both with children, they found weak but significant stress increasing for women (β = 0.24, 95% CI 0.04–0.45).

Jakubowski and Sitko-Dominik [10] carried out a cross-sectional study with a total of 285 participants, exploring the relationship between “distance education” and “teachers’ well-being” and their social relations during the COVID-19 pandemic’s first two waves. In particular, stress levels were estimated by The Depression Anxiety & Stress Scales-21 (DASS-21). They found a weak stress level correlation with the following variables: total number of children (*r* = 0.27, *P* < 0.05), number of children 16–19 years old (*r* = 0.24, *P* < 0.01), relationship quality (*r* = −0.29, *P* < 0.01), and social relation quality (*r* = −0.22, *P* < 0.01). The correlation between stress level and relationship quality during the second wave remains weak while the correlation with social relation quality was moderate (*r* = −0.40, *P* < 0.01). Moreover, General social support (*r* = −0.108), Emotional social support (*r* = −0.09), and Relationship satisfaction (*r* = −0.09) during the first wave showed very weak correlation, unlike the second wave when correlation was weak for general social support (*r* = −0.24, *P* < 0.01), emotional social support (*r* = −0.25, *P* < 0.01), and relationship satisfaction (*r* = −0.25, *P* < 0.01). Finally, for the variables “perceived injustice,” “blame/unfairness,” and “severity/irreparability” authors report no data during the first wave, while in the second wave the “perceived injustice” shows weak value (*r* = −0.36, *P* < 0.001), “blame/unfairness” shows a moderate value (*r* = 0.58, *P* < 0.001) and “severity/irreparability” (*r* = 0.63, *P* < 0.001) show strong value. Finally, multivariate analysis showed that during the first wave the number of children between 9 and 15 years of age explain 6% of the variability in stress levels, whereas during the second wave the variables “relationship quality,” “social support,” “blame/unfairness,” and “severity/irreparability” explain 47%.

Lipert *et al*. [11] conducted a web-based survey with 1959 participants (1681 women and 278 men) during the lockdown to investigate the relationship between stress and sleep quality across different work modalities, also considering physical activity. Stress was measured using the Perceived Stress Scale (PSS).

All the subjects showed a mean moderate stress level in the following categories: “working in the workplace” (21.5 ± 7.1), “working remotely” (21.5 ± 7.2), and “non-working” (22.6 ± 7.5, *P* < 0.001).

Mari *et al*. [12] conducted a web-based survey with 628 participants (489 women and 139 men) to assess stress levels using the PSS questionnaire across different professions, including teachers, practitioners (lawyer, psychologist, accountant, etc.), managers, and executive employees. Overall, the results highlighted a mean low-stress level, even if the teachers (16.94 ± 5.46) had a mean higher level of stress followed by executive employees (16.27 ± 6.23), practitioners (15.89 ± 5.35), and managers (15.17 ± 5.39).

Sandoval-Reyes *et al*. [13] conducted a web-based survey with 1285 respondents (847 women and 438 men) to study the mediating effect of stress in the relationship between remote work demand (RWD) and work-life balance (WLB), productivity (WP), job satisfaction (WS), and engagement (WC). Stress levels were measured using the Work Stress Questionnaire (Folkman and Lazarus’s, 1985). RWD has a direct, positive effect on stress (STR) (β = 0.27, *P* < 0.01), an indirect effect on WLB (β = −0.05, 95% CI −0.07, −0.04, *P* < 0.01), and on WS (β = −0.01; 95% CI −0.11, −0.07, *P* < 0.01) through work stress. Moreover, there is an indirect effect of RWD on WP (β = −0.05; *P* < 0.01; 95%CI −0.07, −0.04) and WC (β = −0.06; *P* < 0.01; 95% CI −0.08, −0.04) through work stress (STR). They found a significant difference in the relation between STR and WP and the multigroup significance test showed a significant value (β = −0.14; *P* < 0.01) when comparing coefficients from the men’s group (β = −0.29; *t* = 5.87; *P* < 0.01) and the women’s group (β = −0.15; *t* = 4.28; *P* < 0.01).

Şentürk *et al*. [3] carried out a cross-sectional, web-based survey with a total of 459 participants, including 205 women and 254 men, to investigate the stress level using the Depression Anxiety Stress Questionnaire—Short Form (DASS-21). Firstly, they reported the frequency of normal stress subjects (369/459, 80.4%), mild stress subjects (89/459, 19.4%), moderate stress subjects (1/459, 0.2%), and no one with high stress level. Finally, they investigated the relationship of different variables on stress level reporting results for university degree or a postgraduate degree subjects (β = −0.02, 95% CI—0.92, 0.46, *P* = 0.51), changes in time spent on household chores (β = 0.01, 95% CI—0.17, 0.21, *P* = 0.85), changes in time spent on childcare (β = −0.02, 95% CI −0.29, 0.17, *P* = 0.61), and leisure-Time Exercise Questionnaire score (β = −0.01, 95% CI –0.02, 0.01, *P* = 0.88).

Truzoli *et al*. [14] conducted a web-based survey with 385 participants, including 226 women and 159 men, to explore the level of risk factors (e.g. stress) and protective factors (e.g. locus of control) and their impact on satisfaction levels during social distancing. The stress was evaluated through Quick stress assessment questionnaire (Valutazione Rapida dello Stress—VRS by Tarsitani and Biondi, 1999). Through the Spearman’s coefficient test they observed a strong linear correlation between stress and depression (Rho 0.78; *P* < 0.00), stress and anxiety (Rho 0.65; *P* < 0.00), conversely, they reported a low linear correlation between stress and locus of control test (Rho 0.39; *P* < 0.00) and finally a negative linear correlation between stress and self-efficacy (Rho −0.36; *P* < 0.00).

Platts *et al*. [8] performed a cross-sectional study with 623 respondents, including 234 women, 384 men, and 5 missing data, to analyze “wellbeing” using different “stress markers” whose results were reported by the authors using the questionnaire Copenhagen Psychosocial Risk Assessment Questionnaire (COPSOQIII). The score showed a moderate level of stress for “cognitive stress” equal to 31 and a low level for “somatic stress” equal to 26.

Chu *et al*. [17] conducted a web-based survey with 500 participants (288 women and 212 men) to investigate how remote working affected stress levels, using three measures: sleep quality, loss of energy, and depressed mood.

Results highlighted that work-life balance was negatively associated with stress level (β = −0.22, *P* < 0.00). Moreover, there was a positive relation (β = 0.63, *P* < 0.00) with the employees’ participation in non-work-related activities during working hours.

### Quality of life

Barbieri *et al*. [16] conducted a web-based survey involving 293 participants, including 216 women and 77 men. The study investigated the impact of “job demands,” “organizational job,” and “personal resources” on workers’ quality of life considering the potential mediating role of job satisfaction and perceived stress. The authors reported an average score of 0, not specifying any range for quality of life. Moreover, they found that stress explained 55.3% and 58.9% of the proportion of variance in the quality of life predicted for remote workers and for women sub-sample, respectively.

Barone *et al*. [18] compared the effect on the “quality of life” before COVID-19 pandemic *vs* the pandemic phase, using the SF-36 questionnaire, on “general health” (70.5 ± 15.1 *vs* 69.9 ± 16.5), “physical functioning” (92.1 ± 14.5 *vs* 91.7 ± 16.0), “role limitations due to physical health” (93.7 ± 22.5 *vs* 88.1 ± 26.4), and “energy/fatigue” (57.6 ± 17.9 *vs* 54.5 ± 19.6, *P* < 0.05) highlighting no significant score changes. Conversely, a significant change in the score was found for “pain” (87.1 ± 14.7 *vs* 81.7 ± 18.1; *P* < 0.00), “emotional well-being” (77.5 ± 14.8 *vs* 71.4 ± 17.9; *P* < 0.00), “social functioning” (90.5 ± 17.4 *vs* 84.1 ± 19.4; *P* < 0.01) and “role limitations due to emotional health” (87.4 ± 26.2 *vs* 74.8 ± 36.2).

Chu *et al*. [17], using all the items adopted by Chaiprasit and Santidhirakul (2011), studied the level of “happiness” which had a high impact on the three variables: “feed joy at work” (4.92 ± 1.58), “satisfied with work” (4.47 ± 1.52) and “enthusiastic at work” (4.01 ± 1.53) related to a moderate “quality of life.”

Girish *et al*. [15] studied the association between “remote working” and workers “quality of life.” The study reported correlations defined as “acceptable” (AVE > 0.5) for “self-efficacy” (AVE 0.60) and “job satisfaction” (AVE 0.57) where AVE stands for Average Variance Extracted, while “communication/collaboration,” “work efficiency,” “autonomy,” “fairness in appraisal,” “work–life balance,” and “workplace creativity” were reported with a level of correlation below the acceptable value (AVE < 0.5).

## Discussion

The results confirm a link between stress and the balance of private/family and work life, impacting the quality of remote work, particularly for women with children who do not share household duties with their partner [3, 7, 9]. Inequality is further emphasized by education levels [20]. Chung *et al*. highlight that remote work flexibility affects domestic responsibilities, with women shouldering most tasks. This imbalance increases stress and reduces productivity, despite the potential for improved work-life balance [21]. Stress increases with age, as shown by Jakubowski and Sitko-Dominik [10]. Research highlights that aging often reduces coping resources. Lachman and Agrigoroaei (2010) noted that age decreases perceived control and stress management, raising the risk of fatigue [22]. This suggests that aging is linked to reduced coping resources and a higher risk of burnout due to increased stress. The relationship between stress and age could suggest that aging is accompanied by a potential decrease in coping resources (which could be the result of cognitive decline) and by burnout, associated with a high level of stress [23].

According to Mari *et al*. [12], teachers are the professional category most exposed to stress. Chu17 found that employees working beyond regular hours experience higher stress levels. Studies suggest that increased stress among teachers may result from adapting to new work methods and learning new communication strategies for distance learning [24]. For employees working overtime, fatigue affects perception, reasoning, judgment, and decision-making, leading to slower reaction times and reduced cognitive abilities, such as logical reasoning and concentration [25].

Lipert *et al*. [11] found higher stress levels in the unemployed compared to remote or in-person workers. Unemployed individuals are already stressed due to job loss, and this may be exacerbated by increased exposure to media during the pandemic, which heightened fear, anxiety, and stress [26]. Numerous studies highlight the increase in stress among teachers and the unemployed, as well as the impact of fatigue on work performance. Sutton and Harper [27] emphasize that teachers experience significantly higher stress levels than other professions, partly due to the need to adapt to new teaching methodologies and technologies. Additionally, Bodner *et al*. [28] finds that the unemployed report higher stress levels than those working remotely or in-person, as the absence of work and exposure to negative news during the pandemic amplify anxiety and fear, further increasing their stress levels. These studies clearly demonstrate how different professional categories experience stress in distinct ways, influenced by contextual and personal factors.

Galanti *et al*. [7] and Jakubowski and Sitko-Dominik [10] found that work autonomy and self-leadership positively impact productivity and work commitment. These resources have supported productivity during the pandemic, benefiting both organizations and employees [29].

Galanti *et al*. [7] also links social isolation to stress, suggesting that improving communication with colleagues and superiors can reduce isolation. Lack of interaction has been shown to slow problem-solving [30]. Mann and Holdsworth found that remote workers often lack social support, leading to insecurity and reduced confidence.

Social interaction at work is considered “absolutely important,” and remote workers often experience stress from being separated from colleagues [31]. Lal and Dwivedi note that to compensate for this lack of interaction, remote workers often seek social connections with family members [32]. Regarding quality of life, Girish *et al*. [15] found that remote work positively impacts self-efficacy, creativity, job satisfaction, and overall quality of life. The flexibility and freedom of remote work can enhance quality of life compared to on-site work.

Barone Gibbs *et al*. [18] highlights how the quality of life can decline for remote workers, who experience pandemic-related effects such as poor sleep, mood changes, concentration difficulties, and work dissatisfaction. Kotova *et al*. and Tanashyan *et al*. also found that anxiety and depression are frequently linked to sleep disorders and reduced sleep quality during the COVID-19 pandemic [33]. Felstead and Henseke [34] highlight that remote work flexibility can improve well-being, though it also brings challenges like reduced social interaction, which the COVID-19 pandemic has worsened. Wang *et al*. [35] examine the impact of the health crisis on workers’ psychological well-being, linking remote work to sleep quality, anxiety, and depression.

Chu *et al*. [17] finds that work enthusiasm and happiness remain stable, thanks to a healthy work-life balance that reduces stress. Awada *et al*. emphasize that work-life balance is crucial for psychological well-being, quality of life, and stress relief [36]. Across the studies, practical strategies for managing stress and promoting well-being in remote work emerge alongside its challenges. Barbieri *et al*. [16] highlight job design—analyzing and modifying the content, structure, and environment of jobs within the social, physical, and organizational context—as a practice for improving job satisfaction. The authors suggest identifying stress sources and then applying job design to address them [37]. Job design is linked to individual, group, and organizational outcomes [38], particularly affecting job satisfaction. Barbieri *et al*. [16] suggest that job design can promote well-being in remote work, especially during crises like the pandemic. Many studies [7–9, 14, 16, 18] emphasize the importance of supporting workers, through feedback, interaction spaces [12, 16], time management autonomy [7], flexible schedules [17], reduced workload [11], and effective management tools [7, 15]. Sandoval-Reyes *et al*. [13] and Şentürk *et al*. [3] also stress the value of professional support, such as that offered by psychologists. In particular, these authors refer to psychological counseling that can be provided remotely [3, 13] and can be supported by informal support groups [13]. Other useful tips for managing stress and promoting well-being are described in terms of trainings sponsored by the organization. In a context of increasing work-related stress and emotional difficulties, the need for professional support has become increasingly evident. Kawachi and Berkman [39] highlight that social networks play a crucial role in promoting mental health, suggesting that strong and meaningful relationships can serve as a buffer against anxiety and depression. These authors argue that solid social support not only provides emotional backing but also offers opportunities to cope with stress more effectively, thereby enhancing psychological resilience [39]. Galanti *et al*. [7] and Platt *et al*. [8] recommend training to enhance leadership and self-leadership skills. Jakubowski and Sitko-Dominik [10] suggest courses focused on problem-solving and emotion regulation, while Mari *et al*. [12] and Truzoli *et al*. [14] advocate for training in stress management and life skills.

All of these studies highlight the importance of organizational support for remote workers, including analyzing and managing stress sources and providing tools (psychological, social, and physical) to improve psychological well-being.

### Limitations

This systematic review has several limitations. Firstly, the authors used different questionnaires with varying scoring methods to assess stress and quality of life, and not all studies provided clear criteria for classification. Secondly, only a few studies presented results stratified by stress levels. Finally, the review included studies with different designs, such as web-based surveys, prospective surveys, and cross-sectional studies.

## Conclusions

This systematic review explored the relationship between remote working, stress levels, and the quality of life during the COVID-19 pandemic, showing its significant impact on health. The review identified predictors (work-family conflict and social isolation) linked to improvements or declines in quality of life/stress. Future efforts should focus on enhancing work-family balance and preventing social isolation.

## Supplementary Material

ckae167_Supplementary_Data

## Data Availability

Upon reasonable request. Key points Nel (2019), a new working method called “remote working” was implemented to address the coronavirus emergency. Remote working can be associated with stress issues, and it can also impact the quality of life. Increasing stress may be due to the blending of personal and professional life. Additionally, certain occupational categories are more susceptible to stress compared to others. Autonomous work and self-leadership are positively associated with productivity and work engagement in the context of “remote working.” Finally, the quality of life is affected by insomnia-related issues.
