# Correction to: Remote workers’ life quality and stress during COVID-19: a systematic review

**DOI:** 10.1093/eurpub/ckaf169

**Published:** 2025-09-19

This is a correction to: Elisabetta Carraro, Paola Rapisarda, Daniela Acquadro Maran, Sofia Filippetti, Marco Palella, Eliana Pellegrino, Margherita Ferrante, Giuseppe La Torre, Maria Fiore, Remote workers’ life quality and stress during COVID-19: a systematic review, *European Journal of Public Health*, Volume 35, Issue 1, February 2025, Pages 141–152, https://doi.org/10.1093/eurpub/ckae167

In the originally published author list of this article, the first and last names of authors Elisabetta Carraro, Paola Rapisarda, Marco Palella and Giuseppe La Torre were inadvertently reversed. This error has been corrected.

