# Supplementary material for: Remote workers’ life quality and stress during COVID-19: a systematic review
Source: Eur J Public Health. 2025 Feb 6;35(1):141–52. doi: 10.1093/eurpub/ckae167 (PMC11832161; doi:10.1093/eurpub/ckae167)
Supplement: ckae167_Supplementary_Data [file ckae167_supplementary_data.zip › ckae167_Supplementary_Data/ejph-2024-01-om-0064-File003.docx]

**APPENDIX A**

**SEARCH STRATEGY OF KEYWORDS**

| **Database** | **Search strategy** |
| --- | --- |
| Pubmed | (administrative workers) OR (technical staff) OR (teachers) OR (smart working) AND (stress);  (work from home) OR (agile work) AND (quality of life);  ((work related health) OR (life satisfaction) OR (well-being) OR (stress) OR (occupational stress) OR (subjective stress) OR (work related stress)) AND ((Covid-19) OR (pandemic) OR (Sars-COV-2)) |
| Scopus | (administrative workers) OR (technical staff ) OR (teachers) AND (smart working);  (working from home) OR (agile work) AND (quality of life);  ((work related health) OR (life satisfaction) OR (well-being) OR (stress) OR (occupational stress) OR (subjective stress) OR (work related  stress)) AND ((covid-19) OR (pandemic) OR (sars-cov-2)) |
| Web of science | administrative workers OR technical staff OR teachers AND smart working;  working from home OR agile work AND quality of life; (work related health OR life satisfaction OR well-being OR stress OR occupational stress OR subjective stress OR work related stress) AND (Covid-19 OR pandemic OR Sars-COV-2) |
