# Supplementary material for: Remote workers’ life quality and stress during COVID-19: a systematic review
Source: Eur J Public Health. 2025 Feb 6;35(1):141–52. doi: 10.1093/eurpub/ckae167 (PMC11832161; doi:10.1093/eurpub/ckae167)
Supplement: ckae167_Supplementary_Data [file ckae167_supplementary_data.zip › ckae167_Supplementary_Data/ejph-2024-01-om-0064-File005.docx]

**APPENDIX B**
**QUALITATIVE EVALUATION OF CASE-CONTROL STUDIES ACCORDING TO THE NEWCASTLE-OTTAWA SCALE: QUALITY ACCESS SCALE FOR CROSS SECTIONAL STUDIES**

|  | **Selection** | | | | **Comparability** | | **Outcome** | | **Total** |
| --- | --- | --- | --- | --- | --- | --- | --- | --- | --- |
| Author, years | Representativeness of the sample | Sample size | Non respondents | Ascertainment of exposure | The study control for the most important factor | The study control for any additional factor | Assessment of the outcome | Statistical test |  |
| Barbieri, 2021 | * | * | * | * | * |  | * | * | 7/10 |
| Barone Gibbs, 2021 | * | * | * | ** | * |  | ** | * | 9/10 |
| Galanti, 2021 | * | * | * | ** | * | * | * | * | 9/10 |
| Graham,2021 | * | * | * | ** | * |  | ** | * | 9/10 |
| Jakubovski, 2021 | * | * | * | * | * |  | ** | * | 8/10 |
| Lipert, 2021 | * | * | * | * | * | * | ** | * | 9/10 |
| Mari, 2021 | * | * | * | ** | * |  | ** | * | 9/10 |
| Sandoval-Reyes, 2021 | * | * | * | * |  |  | ** | * | 7/10 |
| Senturk,2021 | * | * | * | ** | * |  | * | * | 8/10 |
| Truzoli, 2021 | * | * | * |  | * | * | * | * | 7/10 |
| Girish, 2022 | * | * | * | * | * | * | * | * | 8/10 |
| Platts, 2022 | * | * | * | ** | * |  | ** | * | 9/10 |
| Chu, 2022 | * | * | * | * | * |  | * | * | 7/10 |
